# Supplementary material for: Haemoparasitic Infections in Cattle from a Trypanosoma brucei Rhodesiense Sleeping Sickness Endemic District of Eastern Uganda
Source: Trop Med Infect Dis. 2020 Feb 7;5(1):24. doi: 10.3390/tropicalmed5010024 (PMC7157584; doi:10.3390/tropicalmed5010024)
Supplement: Supplementary file 1 [file tropicalmed-05-00024-s001.pdf]

Tropical Medicine and
Infectious Disease

Supplementary Materials

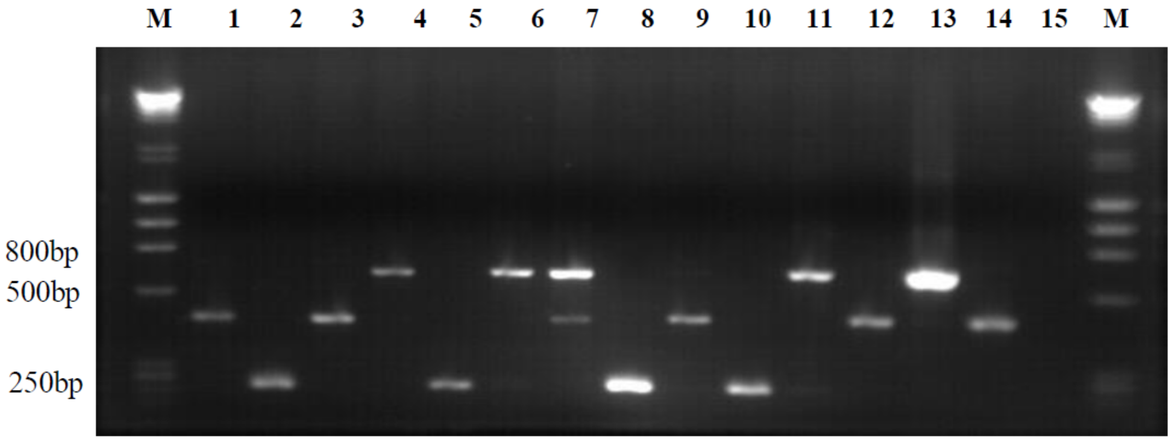

Figure S1. Electrophoretic analysis of ITS PCR products. Representative Agarose gel showing the different sizes of the ITS1 region for the different trypanosome species amplified using non-nested primers. The expected band sizes are; T. brucei—480 bp, T. vivax—250 bp and T. congolense—700 bp. Lanes M—standard DNA marker, Lanes 1–13—cattle samples with successfully amplified ITS1 region. Lane 14—Positive control (T.b.b GVR-35), Lane 15—Negative control (Sterile water).

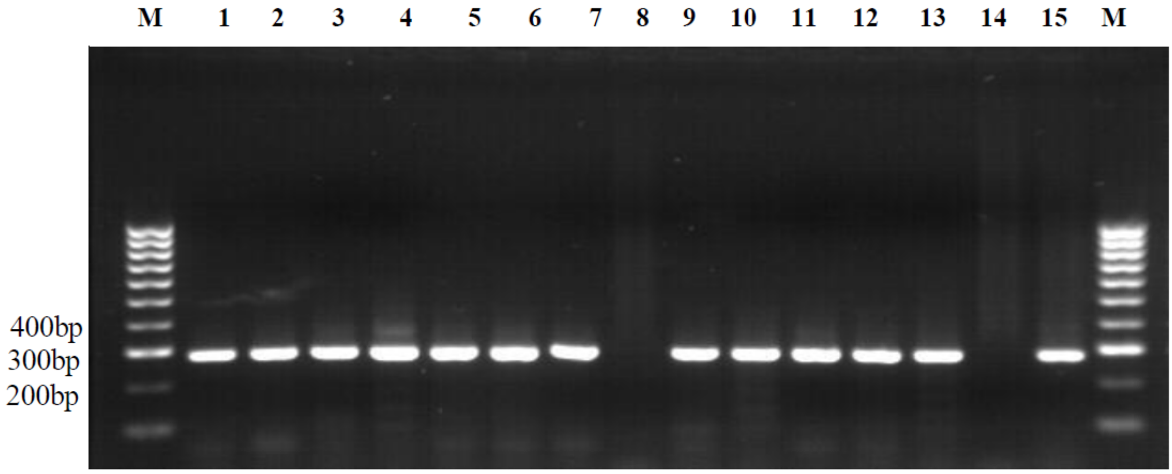

Figure S2. Electrophoretic analysis of SRA PCR products. Representative Agarose gel results showing the amplified SRA gene (280 bp). Lanes M—100 bp standard DNA marker, Lanes 1–7 and 9–13—cattle samples with successfully amplified SRA gene, Lane 8—cattle sample with no amplification of SRA gene. Lane 14—Negative control (Sterile water), Lane 15—Positive control (T.b.r 729).

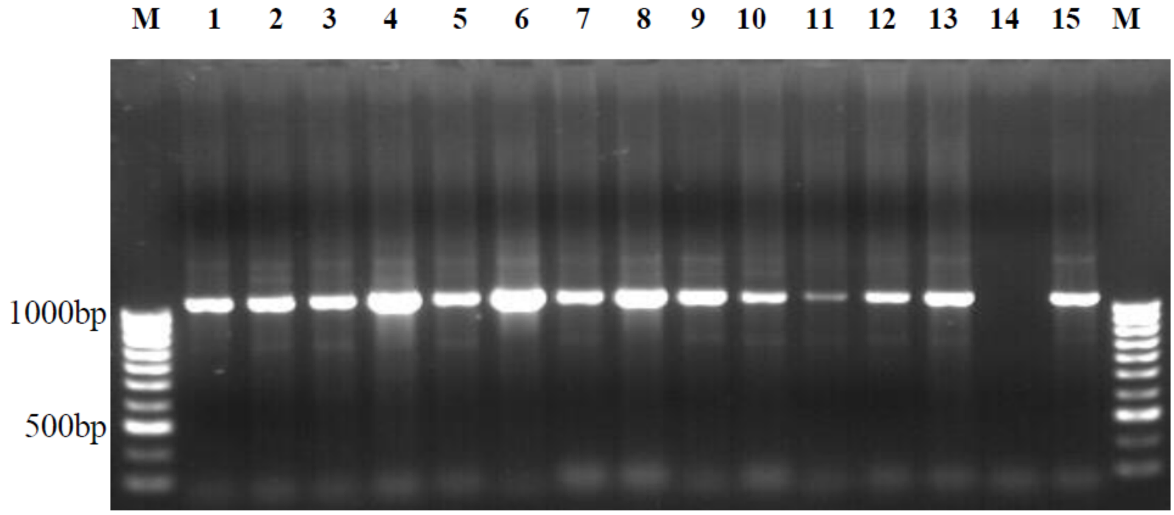

Figure S3. Electrophoretic analysis of Theileria PCR products. Representative Agarose gel results showing the amplified SSU rRNA gene fragment of the Theileria species of expected band size 1098 bp. Lanes M—100 bp standard DNA marker, Lanes 1–13—cattle samples with positive amplifications, Lane 14—Negative control (Sterile water), Lane 15—Positive control (Bovine field isolate confirmed with Theileria species).

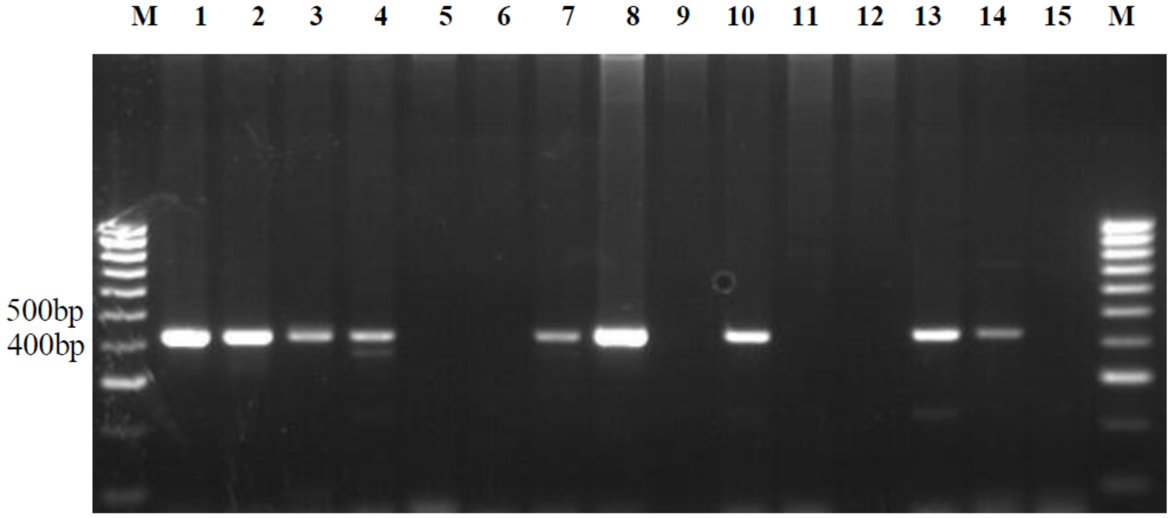

Figure S4. Electrophoretic analysis of Babesia PCR products. Representative Agarose gel results showing the amplified 18S rRNA gene fragment of the Babesia species (422–440 bp). Lanes M—100 bp standard DNA marker, Lanes 1–4, 7–8, 10 and 13—cattle samples with positive amplifications, Lanes 5–6, 9 and11–12—cattle samples with negative amplifications, Lane 14—Positive control (Bovine field isolate confirmed with Babesia species), Lane 15—Negative control (Sterile water).

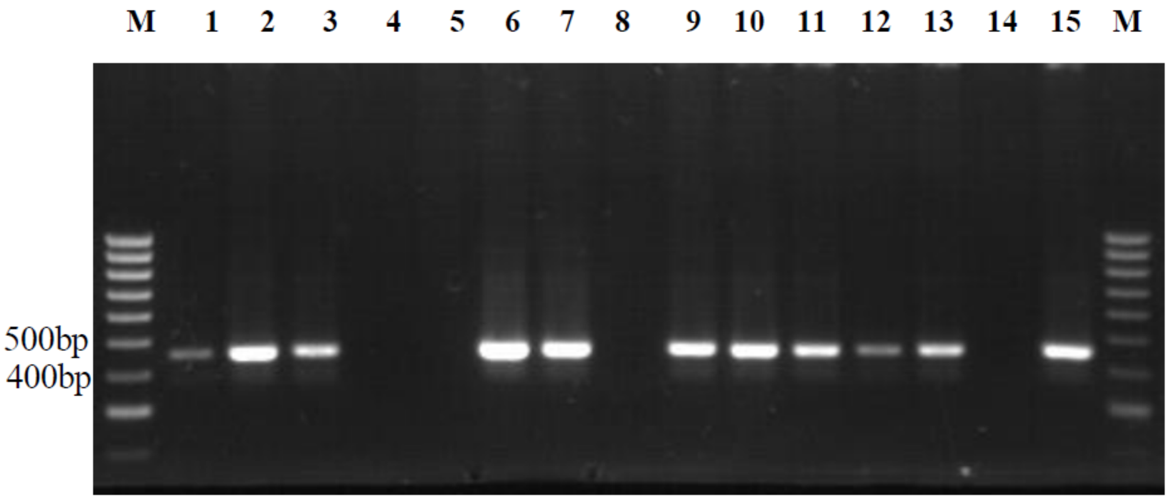

Figure S5. Electrophoretic analysis of Anaplasma PCR products. Representative Agarose gel results showing the amplified 16S rRNA gene fragment of the Anaplasma species; the expected band size is 452 bp. Lanes M—100 bp standard DNA marker, Lanes 1–3, 6–7 and 9–13—cattle samples with positive amplifications, Lanes 4–5 and 8—cattle samples with negative amplifications, Lane 14—Negative control (Sterile water), Lane 15—Positive control (Bovine field isolate confirmed with Anaplasma species).
